# Plasticization Effect of Poly(Lactic Acid) in the Poly(Butylene Adipate–*co*–Terephthalate) Blown Film for Tear Resistance Improvement

**DOI:** 10.3390/polym12091904

**Published:** 2020-08-24

**Authors:** Do Young Kim, Jae Bin Lee, Dong Yun Lee, Kwan Ho Seo

**Affiliations:** Department of Polymer Science and Engineering, Kyungpook National University, Daegu 41566, Korea; ddyykk9655@gmail.com (D.Y.K.); moasi333@gmail.com (J.B.L.)

**Keywords:** poly(lactic acid), poly (butylene adipate–*co*–terephthalate), plasticizer, blown film, tear resistance

## Abstract

The mechanical properties and tear resistance of an ecofriendly flexible packaging film, i.e., poly(lactic acid) (PLA)/poly (butylene adipate–*co*–terephthalate) (PBAT) film, were investigated via a blown film extrusion process. The application of PLA and PBAT in product packaging is limited due to the high brittleness, low stiffness, and incompatibility of the materials. In this study, the effects of various plasticizers, such as adipate, adipic acid, glycerol ester, and adipic acid ester, on the plasticization of PLA and fabrication of the PLA/PBAT blown film were comprehensively evaluated. It was determined that the plasticizer containing ether and ester functionalities (i.e., adipic acid ester) improved the flexibility of PLA as well as its compatibility with PBAT. It was found that the addition of the plasticizer effectively promoted chain mobility of the PLA matrix. Moreover, the interfacial adhesion between the plasticized PLA domain and PBAT matrix was enhanced. The results of the present study demonstrated that the plasticized PLA/PBAT blown film prepared utilizing a blown film extrusion process exhibited improved tear resistance, which increased from 4.63 to 8.67 N/mm in machine direction and from 13.19 to 16.16 N/mm in the transverse direction.

## 1. Introduction

Poly(lactic acid) (PLA) is a well-known biodegradable polymer, which has attracted significant attention as a possible solution to alleviate environmental pollution and waste accumulation. PLA is derived from renewable resources, e.g., from sugars in corn starch or cassava. Moreover, after consumption, it completely decomposes into water and carbon dioxide via composting [1,2,3]. PLA is inexpensive and exhibits remarkable mechanical strength and biocompatibility [4,5]. Nevertheless, its applications have been limited due to some unfavorable characteristics, including low fracture toughness and flexibility, high brittleness, slow crystallization, and narrow processing window. Consequently, the use of PLA in blown film extrusion and foam processes is restricted [6,7]. To improve these properties and enable simple processability in the packaging industry, PLA has been modified by the addition of compatibilizers, such as plasticizers [8,9,10], reactive coupling agents [11,12,13], and fillers [14,15,16]. In addition, blending with other polymers [17,18,19,20], including copolymers and functionalized polymers, has also been reported.

Poly(butylene adipate–*co*–terephthalate) (PBAT) is another commonly known biodegradable polymer. It is derived from petroleum-based resources and exhibits high elongation at break and good ductility. Similarly to thermoplastic elastomers, it also displays low modulus of elasticity [21,22]. However, PBAT is associated with several unfavorable properties, which limit its application. In addition to high cost, the material shows low stiffness and weak tensile strength [23,24]. Thus, a blend of PBAT and PLA with complementary properties has been proposed as one of possible solutions to solve the aforementioned issues. Numerous studies have investigated PLA/PBAT blends [25,26,27,28]. Considering the difference in their solubility parameters, the materials were found to be immiscible and phase separation of their blends is often required [29,30,31]. Hence, the use of PLA/PBAT blends in the film packaging industry is limited because of the undesirable mechanical properties, including poor tear strength due to brittleness of PLA as well as its incompatibility with PBAT. However, these drawbacks could be resolved by the addition of plasticizers to PLA.

Plasticization is a widely employed process to improve the processing behaviors of polymeric materials. Furthermore, plasticization is often used to enhance the original characteristics of polymers, including their flexibility and mechanical and thermal properties, by inducing favorable interactions between the polymer and plasticizer [32]. This method also involves the modification of the intermolecular bonds between the polymer chains, promoting interfacial adhesion and resulting in increased compatibility [33]. Several PLA plasticizers, such as acetyl tributyl citrate [34,35,36], diethyl adipate [37,38], lactides [8,39], and polyethylene glycol [40,41], have been studied. These results have successfully demonstrated the plasticization of PLA by blending with other polymers such as starch and PBAT. However, these studies were focused on a reduced glass transition temperature and the improved elongation at break of PLA.

In this study, we focused on the preparation of plasticized PLA/PBAT blown films utilizing suitable plasticizers to improve tear resistance. The thermal, mechanical, and tear resistance properties as well as the morphology of the plasticized PLA/PBAT blends and films were comprehensively evaluated. Plasticization was carried out to determine the most appropriate plasticizer for PLA. In addition, the effects of the type and content of plasticizers were investigated. It was found that the PBAT blown film containing PLA plasticized by adipic acid ester, which was obtained via a blown process, exhibited improved mechanical and tear resistance properties compared to the PLA/PBAT blown film without any plasticizers.

## 2. Materials and Methods

### 2.1. Materials

PLA (Ingeo™ Biopolymer 4032D) was obtained from NatureWorks LLC (Minnetonka, MN, USA). The melt flow index (MFI) of PLA was determined at 6.0 g/10 min at 190 °C. The load was 2.16 kg and the d-lactic acid content was estimated at 2%. PBAT (EnPol PBG7070) was supplied by Lotte Fine Chemical (Seoul, Korea). Adipate (bis[2–(2–butoxyethoxy)ethyl] adipate) was obtained from Sigma-Aldrich (St. Louis, MO, USA). Adipic acid (EDENOL^®^ 1208) and glycerol ester (LOXIOL^®^ P1141) were purchased from Emery Oleochemicals (Telok Panglima Garang, Malaysia). Adipic acid ester (DAIFATTY^®^-101) was obtained from Daihachi Chemical Industry Co., Ltd. (Osaka, Japan). The aforementioned reagents were used as plasticizers for PLA and their properties are summarized in Table 1. Talc (KCM-6300, KOCH, Seocheon, Korea) and ethylene bis-stearamide wax (HI-LUBE^TM^ bead, Sinwon Chemical, Siheung, Korea) were employed as commercial-grade processing aids to reduce the surface friction of films and promote the usage of antiblocking and nucleating agents.

### 2.2. Preparation of Plasticized PLA Samples

The preliminary experiments were carried out to evaluate the effects of plasticizers on the flexibility of PLA. The PLA pellets were dried in a vacuum oven at 50 °C for 24 h to remove moisture. The weight ratios of the compounds composed of PLA and the plasticizers are summarized in Table 2. The materials were prepared utilizing a Plasti-Corder Lab-Station with a W 50 EHT mixer (Brabender, Duisburg, Germany) equipped with a counter-rotating twin-screw compounder with a bowl volume of 55 cm^3^ and roller blades. The plasticized PLA materials were produced at a barrel temperature of 170 °C with a rotation speed of 50 rpm and residence time of 1 min. After the torque was stabilized, a plasticizer was added at the same temperature and rotation speed, with a residence time of 5 min. The as-prepared compounds were cut into small pieces and shaped into 140 mm × 120 mm rectangles with a thickness of 1 mm using a hydraulic laboratory press (Model 3851, Fred S. Carver Inc., Menomonee Falls, WI, USA). The molding was performed at 170 °C using a preheating time of 4 min at a pressure of 2000 psi for 2 min. The samples were subsequently cooled.

### 2.3. Preparation of Plasticized PLA and PBAT Materials

Based on the results of the preliminary experiments, to plasticize PLA independently, the extrusion was carried out in a counter-rotating twin-screw extruder (TEK 30 MHS, SM Platek, Ansan, Korea) with a 31.6 mm screw diameter (length (L)/diameter (D) = 40) and liquid feeder (SF3141, In Feed Corp., Hwaseong, Korea). Prior to the extrusion, the materials were dried in an oven at 50 °C for 24 h. The extrusion was performed at a screw speed of 300 rpm. The barrel temperatures were set at a profile of 110/160/170/170/160/160/160/160/160 °C with the die temperature set at 160 °C. The extruded strand was cooled in a water bath at room temperature and then dried for 24 h after pelletizing. Prior to extrusion of the plasticized PLA/PBAT blend samples, the components were weighted and mixed mechanically at room temperature. Subsequently, the extrusion was carried out utilizing the same conditions. The components used for the preparation of plasticized PLA and plasticized PLA/PBAT are summarized in Table 3.

### 2.4. Blown Film Process

The samples were prepared using a blown film machine (SJ45-MFG500, Seojin Industry, Incheon, Korea) with a 45 mm single screw and an L/D ratio of 30. The barrel temperatures were set at a profile of 145/145/145/140 °C and the annular die temperature was set at 140 °C. To compare the mechanical properties of the materials, the blown film process conditions were fixed at a blow up ratio of 2.8 and film thickness of 30 µm. Concurrently, the annular die diameter of 80 mm and die gap of 1.2 mm were employed.

### 2.5. Instrumentation and Equipment

Validation of the plasticized PLA samples was performed using an MFI tester (MFI 10, Davenport, Hampshire, UK). The MFI tester was employed to measure the processability of plasticized PLA at 190 °C with a 2.16 kg load. The samples were cut every 30 s, and average values of five measurements were used. The glass transition temperature (*T*_g_), crystallization temperature (*T*_c_), cold crystallization temperature (*T*_cc_), melting temperature (*T*_m_), heat flow of crystallization (Δ*H*_c_), cold crystallization (Δ*H*_cc_), change in enthalpy of melting (Δ*H*_m_), and degree of crystallinity (*X*_c_) of the samples were determined by differential scanning calorimetry (DSC; Q2000, TA instruments, New Castle, DE, USA). The samples were first heated to 200 °C at a rate of 10 °C/min and then kept for 3 min under a nitrogen atmosphere to eliminate the effects of previous thermal history. Subsequently, the samples were cooled and heated again from 20 to 200 °C at the rate of 10 °C/min. The value of *X*_c_ was calculated according to Equation (1):

(1) $$X_{c}\left(\%\right)=\frac{\Delta H_{m}-\Delta H_{cc}}{\Delta H_{m,100}}\;\times \;100,$$

where Δ*H*_m,100_ denotes the heat of melting of 100% crystalline PLA (93.7 J/g) [42].

The dynamic mechanical analysis (DMA) of the samples was conducted employing a dynamic mechanical analyzer (N535, Perkin-Elmer, Boston, MA, USA). The measurements were performed at temperatures from −10 to 100 °C at a heating rate of 5 °C/min and a frequency of 1 Hz under nitrogen atmosphere. The samples with dimensions of 30 mm × 10 mm × 1 mm were prepared utilizing a hydraulic laboratory press.

The morphologies of the film samples were investigated using field-emission scanning electron microscopy (FE-SEM; SU8220, Hitachi, Tokyo, Japan) with an acceleration voltage of 5 kV. The samples were cryogenically fractured in liquid nitrogen and sputtered with Pt for 60 s.

Subsequently, the mechanical properties and tear strength of the blown film samples were evaluated using a universal testing machine (UTM; LR5K Plus, Lloyd Instruments, West Sussex, UK) with a load cell of 500 N and an Elmendorf tear tester (ElmaTear 855, James H. Heal & Co. Ltd., Halifax, UK). Furthermore, the tensile properties and tear strength were analyzed according to the ASTM D882 and ASTM D1922 standards. To obtain an average value, five overlapped film samples were prepared and tested in the machine direction (MD) and transverse direction (TD) for each group.

## 3. Results

### 3.1. Effects of PLA Plasticizer Type and Content on Processability

**Figure 1:**
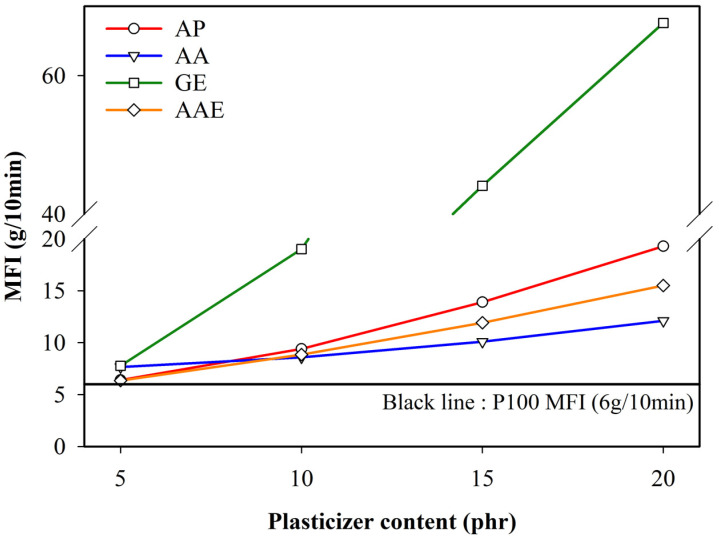
Variation of melt flow index (MFI) for neat PLA and PLA plasticized with varying plasticizer types and contents.

The flow behavior of melted polymers is important in polymer manufacture and processing, including the production of blown and cast films as well as foaming extrusion. The flow behavior of polymers is influenced by various factors, such as the temperature, molecular weight, molecular weight distribution, and addition of fillers and plasticizers [43,44]. The flow behavior can be evaluated by observing the MFI [45]. As a single point test, MFI is a remarkably sensitive technique for distinguishing a change in polymer additives. In the present study, MFI measurements were conducted to investigate the effects of plasticizers on the shear viscosity of PLA. Figure 1 shows the variation of MFI for the plasticized PLA samples with increasing plasticizer content. Evidently, the value of MFI increased with increasing plasticizer amount. It is noteworthy that glycerol ester also acted as a lubricant, which was demonstrated by a sharp increase in MFI (up to 67) compared to other plasticizers. In addition, the sharp increase in MFI could be ascribed to the decomposition of PLA as well as the incompatibility between PLA and the plasticizer. These results showed that glycerol ester was not an adequate plasticizer for the blown film process. Furthermore, an increase in the content of adipate, adipic acid, and adipic acid ester resulted in a gradual increase in the MFI values of the plasticizers. This result was attributed to the molecules permeating the intermolecular spaces between the PLA chains, increasing the mobility of the chain segment and facilitating the flow of PLA [46,47]. Thus, the materials could be effectively applied in blown film processes by controlling their content and type.

### 3.2. Effects of PLA Plasticization on the Thermal Properties

**Figure 2:**
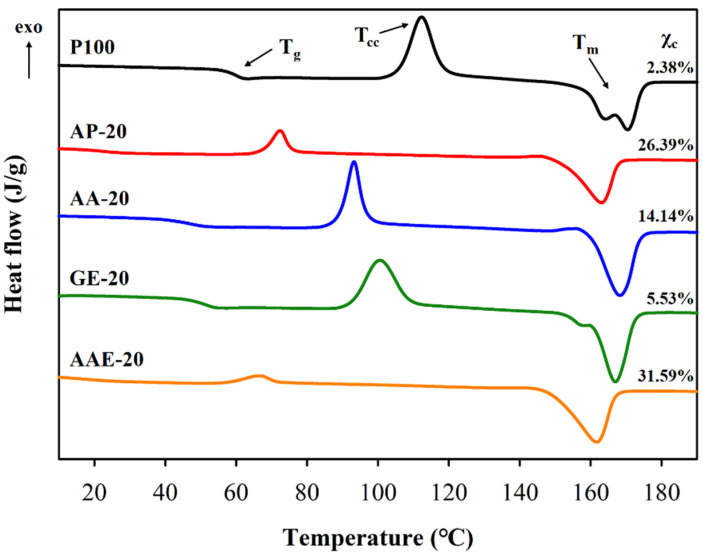
Differential scanning calorimetry (DSC) curves for neat PLA and PLA plasticized with varying plasticizer types and 20 phr.

We subsequently investigated the effects of the plasticizer type and content by DSC to confirm the thermal behavior of the plasticized PLA. Figure 2 shows the secondary heating DSC curves for the plasticized PLA samples, depending on the type of plasticizer with 20 phr. Moreover, the results of DSC measurements for all samples are summarized in Table 4 and Figure S1. As shown in Figure 2, the neat PLA (P100) sample exhibited a transition curve (*T*_g_), cold crystallization peak (*T*_cc_), and a double melting peak (*T*_m_); however, no obvious crystallization peak (*T*_c_) was detected. This is because PLA is a semicrystalline polymer with a slow rate of crystallization. It arises from a semirigid backbone and displays low chain mobility due to the short length of the repeating unit [48,49]. Notably, *T*_g_ and *T*_cc_ of all plasticized PLA samples decreased upon the addition of the plasticizer. In particular, the addition of adipate and adipic acid ester resulted in lower peaks than those observed for other plasticizers because adipate and adipic acid ester exhibit lower molecular weights and contain ether and ester moieties, which are compatible with the ester functionalities in the PLA structure. In addition, the presence of one melting peak and the increase of *X*_c_ for all analyzed samples correlated with the increase in the adipate and adipic acid ester content. Particularly, the *X*_c_ value for AAE-20 increased from 2.38% to 31.59%. This could be related to the increased crystallization rate of PLA, which was a consequence of enhanced chain mobility owing to good compatibility between the plasticizer and PLA [50,51]. Hence, the plasticizers assisted free movement of the chains, which led to more ordered chain packing in the crystalline lattices of PLA. Accordingly, the formation of the crystallization peaks (*T*_c_) of AP-15, AP-20, AAE-15, and AAE-20 was observed in the first cooling curves (Figure S2).

### 3.3. Dynamic Mechanical Properties of Plasticized PLA

**Figure 3:**
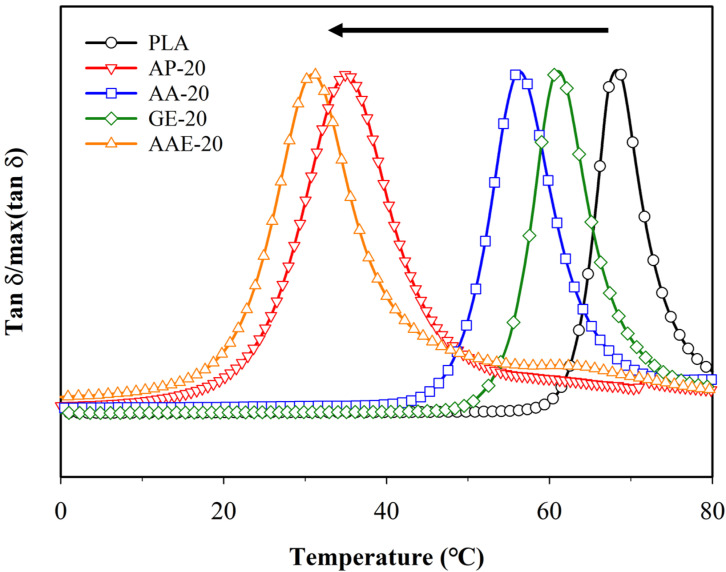
Variation of tanδ with temperature for neat PLA and PLA plasticized with varying plasticizer types and 20 phr.

To further investigate the plasticization of PLA, we subsequently conducted DMA. The results of the analysis for PLA with various plasticizer types and content are presented in Figure 3 and Figure S3, and Table S1. The tanδ peak, defined as the ratio of the loss modulus to the storage modulus with temperature, was related to the *T*_g_ of polymer [52]. The value of the tanδ peak, which was indicated by the *T*_g_ of P100, was observed at 68.8 °C. Notably, tanδ gradually shifted to a lower temperature upon the addition of plasticizers. For the adipic acid ester, tanδ shifted with increasing content of the plasticizer until 31.3 °C. Moreover, similar tendency was observed for adipate. On the other hand, the remaining plasticizers did not cause a significant change in tanδ. This result was in good agreement with previous findings on PLA plasticized by adipate and adipic acid ester. Furthermore, as it can be observed in Figure S3 and Table S1, the decrease in the tanδ values was a consequence of the increase in the free volume and chain mobility of PLA with the increasing plasticizer content. As previously discussed, we confirmed that the plasticization effects of adipic acid ester resulted in improvement of the PLA brittleness. Thus, this plasticizer was deemed as the most suitable for plasticization of PLA. The plasticizer content was appropriated up to 10 phr in consideration of the processability and plasticization of PLA.

### 3.4. Effects of the Plasticized PLA on the Morphology of the Blends with PBAT

**Figure 4:**
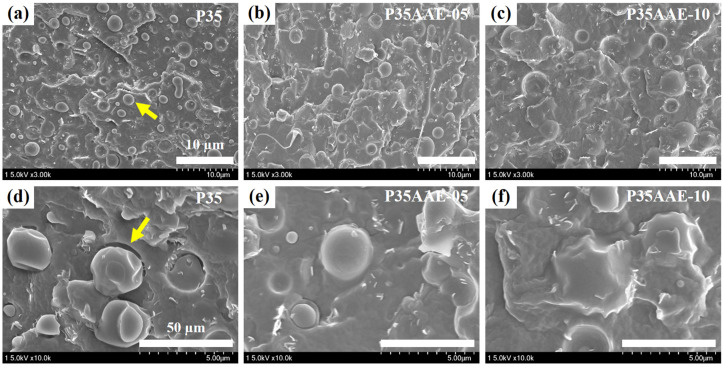
SEM micrographs of the fractured surfaces of the PLA/PBAT and plasticized PLA/PBAT blends at low (upper, scale bar: 10 µm) and high (lower, scale bar: 50 µm) magnifications: (**a**,**d**) P35, (**b**,**e**) P35AAE-05, and (**c**,**f**) P35AAE-10.

Generally, polymer blends can be classified as one phase or phase-separated structures, such as sea-island and co-continuous morphologies. The sea-island morphology is associated with poor mechanical properties, which arise from weak interfacial adhesion between the phases as well as stress at the interface boundaries [53,54]. To improve the phase-separated structures and enhance the mechanical properties of PLA/PBAT blends, SEM was used to investigate the morphology of plasticized PLA in a PBAT matrix. Figure 4 shows the SEM images of the fracture surfaces of PLA/PBAT as well as PLA/PBAT blends plasticized using adipic acid ester. As it can be seen, a sea-island structure containing spherical particles of PLA was observed for P35. Non-uniform dispersion in the PBAT matrix with spherical PLA particles was also observed. Moreover, interfacial debonding between the spherical particles and the matrix were noted. On the other hand, the PLA/PBAT blends plasticized by the addition of adipic acid ester exhibited a smooth surface morphology. It was also found that the voids between the interfaces faded. Notably, the disappearance of the voids between PLA and PBAT increased with increasing plasticizer content. The interfacial adhesion between the PLA domain and the PBAT matrix improved and formed a continuous phase when plasticized PLA was used. The obtained results indicated that adipic acid ester acted as a plasticizer and modified the interfacial adhesion at the phase boundaries. Hence, the plasticizer could affect the tear resistance of the film.

### 3.5. Effects of the Plasticized PLA on the Mechanical Properties of the PBAT Blown Film

**Figure 5:**
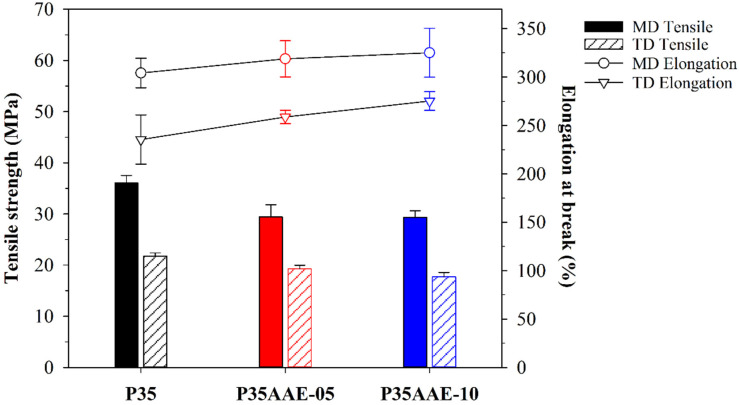
Mechanical properties of the neat PLA/PBAT and plasticized PLA/PBAT blown film.

In a blown film process, the biaxial stretching in MD and TD occurs simultaneously, orienting the polymer chains of the blown film toward the plane of the film. In the present study, the blown films were quenched by cool air, which led to the crystallization and immobilization of the biaxial oriented chains [55,56]. Hence, the biaxial stretching and quenching processes could affect the mechanical properties, such as tensile strength and flexibility, as well as tear resistance. The changes in the tensile strength, elongation at break, and Young’s modulus of the PBAT blown film containing plasticized PLA are summarized in Figure 5 and Table 5. Overall, the tensile strength, elongation at break, and Young’s modulus in MD were higher than those in TD due to more oriented structures resulting from high stresses during the take-up winder process in MD. It is noteworthy that the tensile strength of the PBAT blown film containing plasticized PLA decreased with increasing content of the adipic acid ester plasticizer. In contrast, the elongation at break increased in both MD and TD. In particular, the tensile strength in MD decreased more than in TD. On the other hand, the change in the Young’s modulus showed a different tendency, i.e., it decreased in MD and increased in TD. These results could be attributed to reducing the intermolecular interactions as well as the increase in the chain mobility of PLA because, the orientations of the PLA structures were modified due to the permeation of the adipic acid ester plasticizer into the PLA chains. In addition, the different tendency in the Young’s modulus of MD and TD could be attributed to increase in the crystallinity. The Young’s modulus in high-oriented MD direction is decreased by restricting the orientation of the film, whereas the Young’s modulus in low-oriented TD direction is increased by increasing the crystallinity [57,58,59]. Consequently, the addition of the adipic acid ester plasticizer in the PLA/PBAT blown film affected lower tensile strength, higher elongation at break and the different tendency in the Young’s modulus of MD and TD.

### 3.6. Influence of Plasticized PLA in the PBAT Blown Film of Tear Resistance

**Figure 6:**
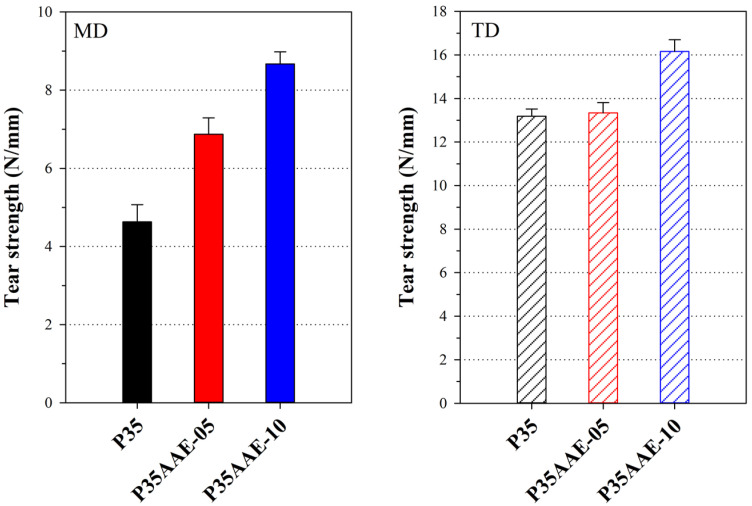
Tear strength of the neat PLA/PBAT and the plasticized PLA/PBAT blown film.

In the polymer fracture mechanism, the voids and interfacial debonding are affected by the non-linearity in the stress–strain relations of the polymer composites. In particular, the interfaces in the polymer blends result in easy fracturing at the interface rather than in the matrix, resulting in low fracture energy [60,61,62]. Figure 6 and Table 5 show the tear strength results obtained for the PBAT blown film with neat PLA and plasticized PLA in MD and TD. The tear strength was considerably higher in TD than in MD as a consequence of the high-orientation of the film. The outcomes revealed that the tear strength of the PBAT film containing plasticized PLA was higher than the pure PLA/PBAT film. Specifically, the tear strength increased from 4.63 to 8.67 N/mm in MD (187%) and from 13.19 to 16.16 N/mm in TD (122%). As mentioned earlier, the presence of plasticized PLA enhanced the interfacial adhesion with PBAT, resulting in the reduction of defects such as voids. In addition, the plasticized PLA/PBAT blend has more homogeneous, because the plasticizer was preferentially located in amorphous phases than crystalline phases and increased the free volume [63,64,65]. Thus, this improvement can be attributed to disperse and absorb the energy required to propagate a crack or a tear. In other words, this indicates that tear resistance improvement is considerably affected by the change in the morphology of the interface than crystallinity. However, the change in the tear resistance of MD and TD revealed a different tendency, because the increase in crystallinity restricted the MD orientation of the film [66]. As can be seen from Figure 6, the change in the tear resistance of MD, which is considerably stretched by the take-up winder, is large.

## 4. Conclusions

In this study, plasticizers with various chemical structures, including adipate, adipic acid, glycerol ester, and adipic acid ester, were used for plasticization of PLA to improve the tear resistance of a PLA/PBAT blown film. The thermal and dynamic mechanical properties of plasticized PLA were comprehensively investigated. We demonstrated that adipic acid ester effectively promoted the chain mobility of the PLA matrix. Thus adipic acid ester containing ether and ester functionalities and exhibiting low molecular weight was deemed as the most suitable plasticizer for PLA. Furthermore, a blend of plasticized PLA and PBAT was prepared by twin-screw extrusion, affording a plasticized PLA/PBAT blown film. Plasticized PLA was found to have a remarkable effect on the morphology, mechanical properties, and tear resistance of the film. The plasticized PLA/PBAT film displayed improved tear resistance of approximately 187% (MD) and 122% (TD) compared to neat PLA/PBAT films. The presence of plasticized PLA in PBAT resulted in the formation of a continuous phase by improving the interfacial adhesion between the PLA domain and the PBAT matrix. In conclusion, we demonstrated that the plasticizer type and content affects the thermal and mechanical properties as well as the processability of PLA. Importantly, the tear resistance of the plasticized PLA/PBAT blown films was significantly influenced by the plasticization of PLA. The results of the present study provide a platform for application of PLA/PBAT blow films as potential biopolymers for the development of ecofriendly packaging films.

## Figures and Tables

**Table 1:** Basic properties of the plasticizers used in this study.

Type (Description)	Product Name	Molecular Weight (g/mol)	Density (g/cm^3^)	Viscosity (mPa·s)	Supplier
Adipate (AP)	Bis[2–(2–butoxyethoxy) ethyl] adipate	434.6	1.01	20.5	Sigma-Aldrich
Adipic acid (AA)	EDENOL^®^ 1208	-	1.03	650–750	Emery Oleochemicals
Glycerol ester (GE)	LOXIOL^®^ P 1141	-	0.93	90–110	Emery Oleochemicals
Adipic acid ester (AAE)	DAIFATTY^®^-101	338	1.10	19	Daihachi Chemical Industry

**Table 2:** Compositions of the plasticized PLA compounds.

Description	PLA (wt %)	AP (phr)	AA (phr)	GE (phr)	AAE (phr)
P100	100	-	-	-	-
AP-05	100	5	-	-	-
AP-10	100	10	-	-	-
AP-15	100	15	-	-	-
AP-20	100	20	-	-	-
AA-05	100	-	5	-	-
AA-10	100	-	10	-	-
AA-15	100	-	15	-	-
AA-20	100	-	20	-	-
GE-05	100	-	-	5	-
GE-10	100	-	-	10	-
GE-15	100	-	-	15	-
GE-20	100	-	-	20	-
AAE-05	100	-	-	-	5
AAE-10	100	-	-	-	10
AAE-15	100	-	-	-	15
AAE-20	100	-	-	-	20

**Table 3:** Compositions of the plasticized PLA/PBAT compounds.

Description	PLA (wt %)	AAE-05 (wt %)	AAE-10 (wt %)	PBAT (wt %)	Talc (phr)	Wax (phr)
P35	35	-	-	65	3	0.3
P35AAE-05	-	35	-	65	3	0.3
P35AAE-10	-	-	35	65	3	0.3

**Table 4:** DSC data for neat PLA and PLA plasticized with varying plasticizer types and contents.

Description	*T*_g_ (°C)	*T*_c_ (°C)	*T*_cc_ (°C)	*T*_m_ (°C)	Δ*H*_c_ (J/g)	Δ*H*_cc_ (J/g)	Δ*H*_m_ (J/g)	*X*_c_ (%)
P100	60.18	-	112.32	170.43	-	33.56	35.79	2.38
AP-05	50.04	-	96.81	168.34	-	27.25	33.96	7.16
AP-10	39.48	-	86.15	165.84	-	21.63	36.54	15.91
AP-15	28.86	78.71	77.04	164.63	3.51	12.67	35.27	24.12
AP-20	-	76.23	72.34	163.06	5.08	9.27	33.97	26.39
AA-05	52.96	-	102.19	169.86	-	27.29	31.76	4.77
AA-10	46.49	-	104.68	169.67	-	26.06	34.07	8.55
AA-15	45.30	-	90.54	167.95	-	21.59	35.21	14.54
AA-20	47.70	-	93.21	168.34	-	20.68	33.93	14.14
GE-05	55.01	-	101.90	168.74	-	26.85	31.53	4.99
GE-10	52.98	-	100.84	168.11	-	23.82	28.64	5.14
GE-15	52.30	-	103.18	167.76	-	29.17	33.73	4.87
GE-20	51.28	-	100.58	166.94	-	25.55	30.73	5.53
AAE-05	49.43	-	95.98	167.84	-	24.83	32.83	8.54
AAE-10	42.55	-	89.09	165.83	-	21.48	32.83	12.11
AAE-15	32.98	81.10	81.43	163.78	3.15	15.88	33.01	18.28
AAE-20	-	74.26	66.92	161.80	12.40	3.83	33.07	31.59

**Table 5:** Mechanical properties and tear strength of the neat PLA/PBAT and plasticized PLA/PBAT blown film.

Description	Direction	Tensile Strength (MPa)	Elongation at Break (%)	Young’s Modulus (MPa)	Tear Strength (N/mm)
P35	MD	36.08 ± 1.44	304 ± 15.2	1782 ± 752	4.63 ± 0.44
	TD	21.77 ± 0.63	235 ± 25.4	528 ± 140	13.19 ± 0.33
P35AAE-05	MD	29.40 ± 2.42	318 ± 18.6	1411 ± 577	6.87 ± 0.42
	TD	19.29 ± 0.67	258 ± 6.9	635 ± 268	13.34 ± 0.47
P35AAE-10	MD	29.32 ± 1.31	325 ± 25.2	1332 ± 520	8.67 ± 0.31
	TD	17.74 ± 0.84	275 ± 9.7	829 ± 167	16.16 ± 0.54

## Supplementary Materials

The following are available online at https://www.mdpi.com/2073-4360/12/9/1904/s1, Figure S1: DSC thermograms of neat PLA and PLA plasticized with different plasticizer types and contents: (a) AP, (b) AA, (c) GE, and (d) AAE, Figure S2: DSC cooling curves of P100, AA-15, AA-20, AAE-15, and AAE-20, Figure S3: Variation of tanδ with varying plasticizer types and contents for neat PLA and PLA plasticized with (a) AP, (b) AA, (c) GE, and (d) AAE, Table S1. DMA data of neat PLA and PLA plasticized with different plasticizer types and contents.
