# Supplementary material for: Plasticization Effect of Poly(Lactic Acid) in the Poly(Butylene Adipate–co–Terephthalate) Blown Film for Tear Resistance Improvement
Source: Polymers (Basel). 2020 Aug 24;12(9):1904. doi: 10.3390/polym12091904 (PMC7564878; doi:10.3390/polym12091904)
Supplement: Supplementary file 1 [file polymers-12-01904-s001.pdf]

Plasticization Effect of Poly(Lactic Acid) in the Poly(Butylene Adipate–*co*–Terephthalate) Blown Film for Tear Resistance Improvement

Do Young Kim, Jae Bin Lee, Dong Yun Lee*, and Kwan Ho Seo*

Department of Polymer Science and Engineering, Kyungpook National University, Daegu 41566, Republic of Korea; ddyykk9655@gmail.com (D.Y.K.); moasi333@gmail.com (J.B.L.)

*Correspondence: dongyunlee@knu.ac.kr (D.Y.L.), khseo@knu.ac.kr (K.H.S.)

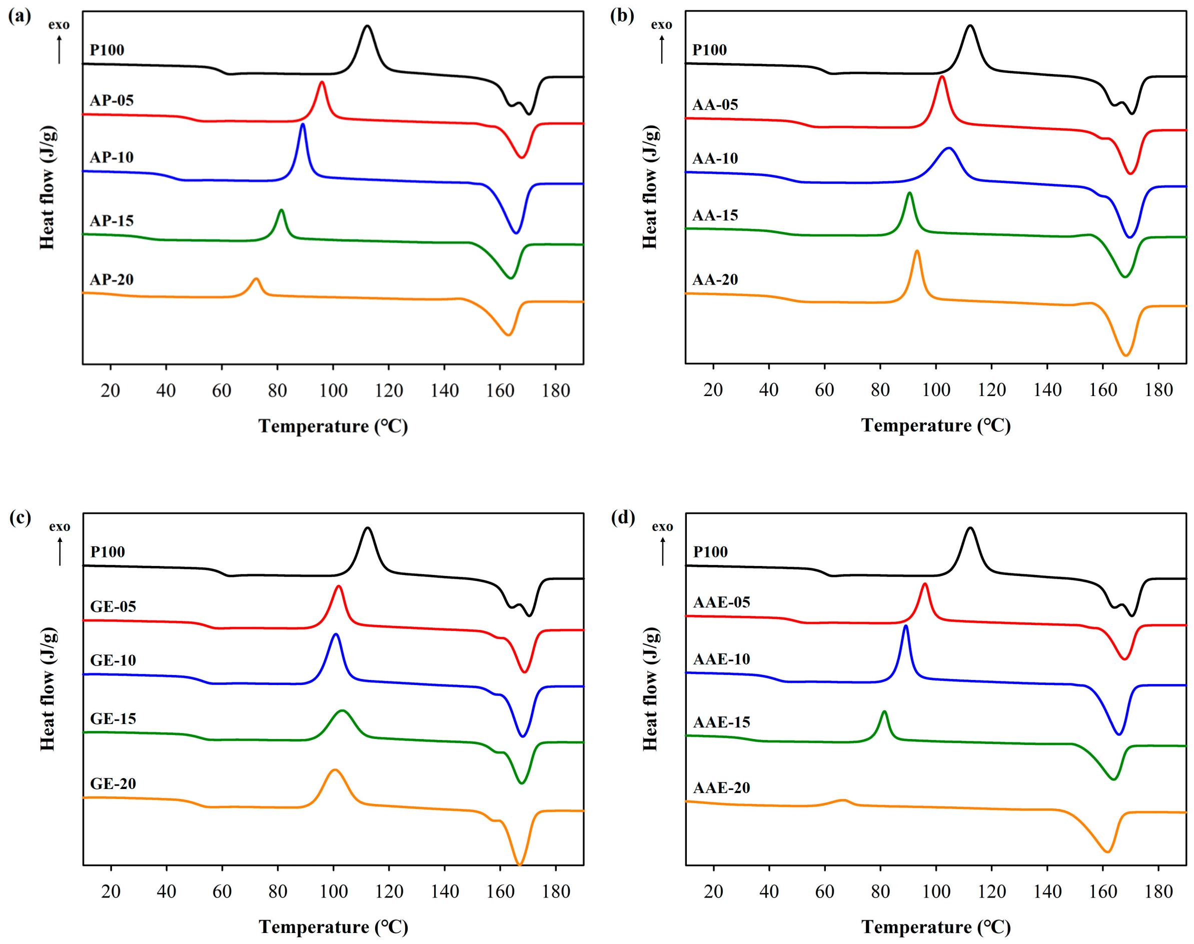

Figure 1. DSC thermograms of neat PLA and PLA plasticized with different plasticizer types and contents: (a) AP, (b) AA, (c) GE, and (d) AAE.

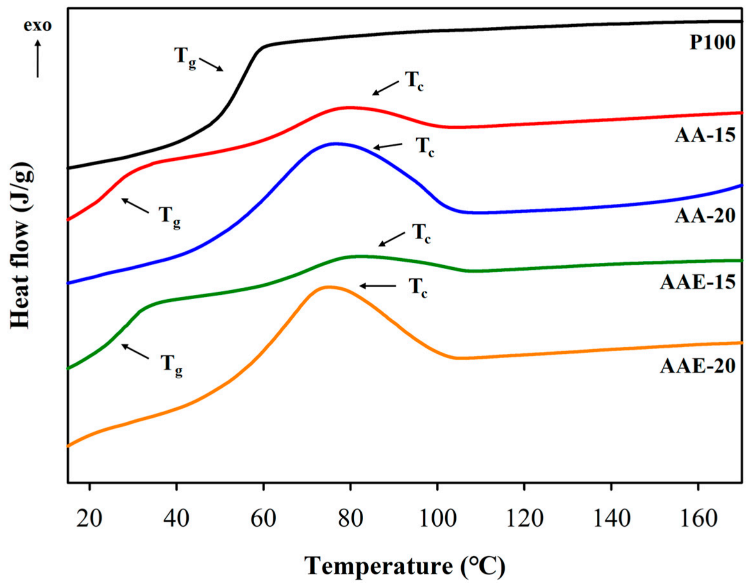

Figure S2. DSC cooling curves of P100, AA-15, AA-20, AAE-15, and AAE-20.

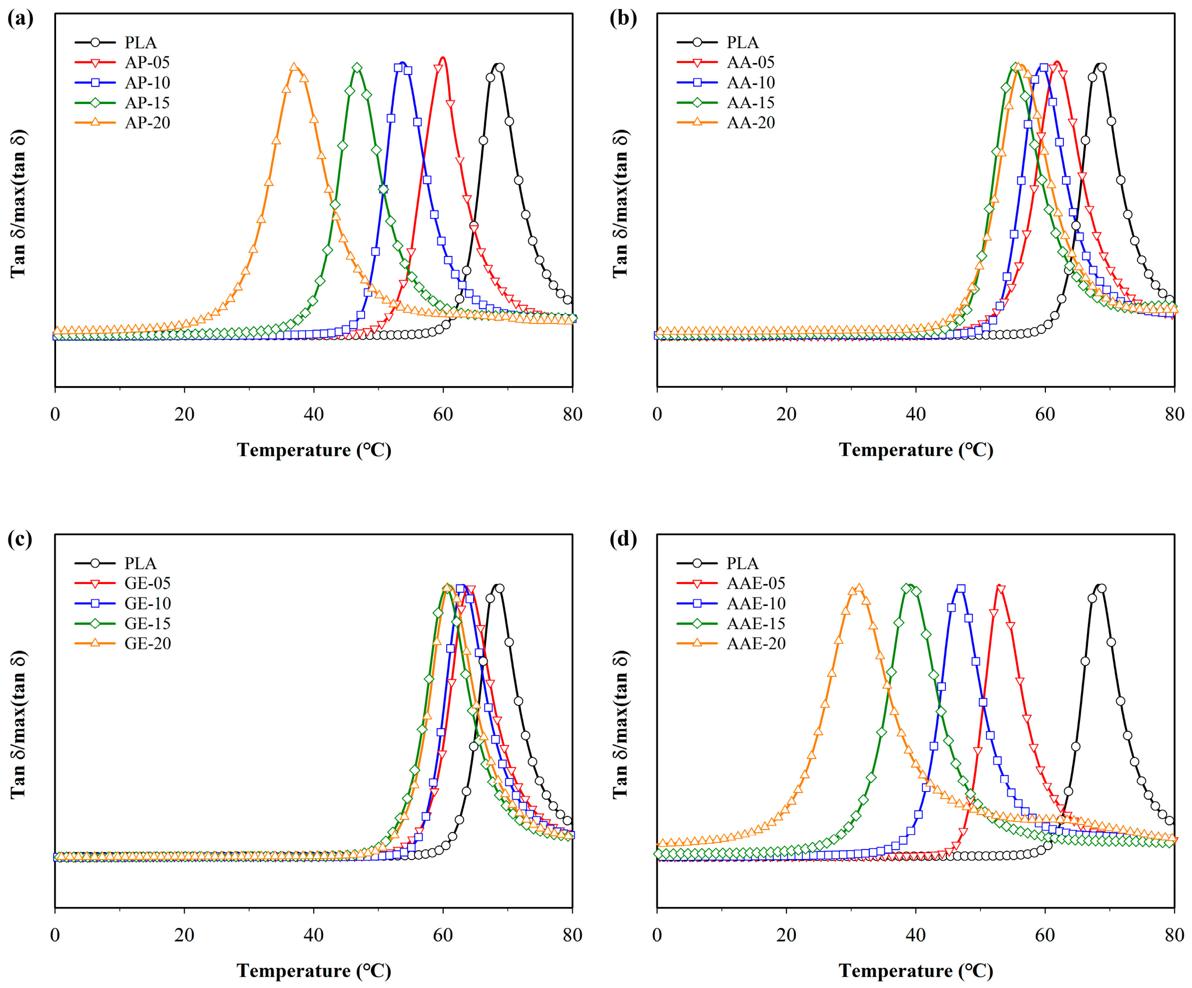

Figure S3. Variation of tan δ with varying plasticizer types and contents for neat PLA and PLA plasticized with (a) AP, (b) AA, (c) GE, and (d) AAE.

Table S1. DMA data of neat PLA and PLA plasticized with different plasticizer types and contents.

Description	T_g (°C)
P100	68.78
AP-05	59.22
AP-10	53.04
AP-15	46.68
AP-20	34.91
AA-05	61.21
AA-10	59.78
AA-15	55.84
AA-20	55.40
GE-05	64.34
GE-10	62.63
GE-15	60.68
GE-20	60.58
AAE-05	53.19
AAE-10	47.02
AAE-15	38.52
AAE-20	31.25
